# Serotonin transporter availability in adults with autism—a positron emission tomography study

**DOI:** 10.1038/s41380-020-00868-3

**Published:** 2020-08-26

**Authors:** Max Andersson, Ämma Tangen, Lars Farde, Sven Bölte, Christer Halldin, Jacqueline Borg, Johan Lundberg

**Affiliations:** 1 Department of Clinical Neuroscience, Center for Psychiatry Research, Karolinska Institutet and Region Stockholm, Stockholm, Sweden; 2 Department of Women’s and Children’s Health, Karolinska Institutet, Center of Neurodevelopmental Disorders (KIND), Center for Psychiatry Research, Stockholm Health Care Services, Region Stockholm, Stockholm, Sweden; 3 Child and Adolescent Psychiatry, Stockholm Health Care Services, Region Stockholm, Stockholm, Sweden; 4 Curtin Autism Research Group, School of Occupational Therapy, Social Work and Speech Pathology, Curtin University, Perth, WA Australia

**Keywords:** Neuroscience, Autism spectrum disorders

## Abstract

Impairments in social interaction and communication, in combination with restricted, repetitive behaviors and interests, define the neurodevelopmental diagnosis of autism spectrum disorder (ASD). The biological underpinnings of ASD are not well known, but the hypothesis of serotonin (5-HT) involvement in the neurodevelopment of ASD is one of the longest standing. Reuptake through the 5-HT transporter (5-HTT) is the main pathway decreasing extracellular 5-HT in the brain and a marker for the 5-HT system, but in vivo investigations of the 5-HTT and the 5-HT system in ASD are scarce and so far inconclusive. To quantify possible alterations in the 5-HT system in ASD, we used positron emission tomography and the radioligand [^11^C]MADAM to measure 5-HTT availability in the brain of 15 adults with ASD and 15 controls. Moreover, we examined correlations between regional 5-HTT availability and behavioral phenotype assessments regarding ASD core symptoms. In the ASD group, we found significantly lower 5-HTT availability in total gray matter, brainstem, and 9 of 18 examined subregions of gray matter. In addition, several correlations between regional 5-HTT availability and social cognitive test performance were found. The results confirm the hypothesis that 5-HTT availability is lower in the brain of adult individuals with ASD, and are consistent with the theory of 5-HT involvement in ASD neurodevelopment. The findings endorse the central role of 5-HT in the physiology of ASD, and confirm the need for a continued investigation of the 5-HT system in order to disentangle the biology of ASD.

## Introduction

Autism spectrum disorder (ASD) is a heterogenic neurodevelopmental condition defined by impairments in social interaction and communication, alongside restricted, repetitive behaviors and interests [1]. While the diagnosis of ASD is categorical, autistic traits are dimensional, with ASD representing a combination of traits in the extreme end of a spectrum [2, 3]. Recent studies suggest high heritability [4], and a prevalence between 1 and 2.5% [5–7]. There is no pharmacological intervention targeting the core symptoms of ASD, and the limited understanding of the biology of ASD hampers the development of such interventions.

In the majority of individuals with ASD, the genetic contribution is driven by a multitude of common gene variants, displayed in a substantial phenotypical variability, while rare penetrable variants lead to the less common syndromic forms with a more homogeneous phenotype [8]. Since multiple etiological pathways of ASD are expected, a thorough characterization and stratification of individuals sampled is needed to disentangle the different possible mechanisms contributing to autism core symptoms [9]. The high prevalence of comorbidity presents a particular challenge in clinical studies on the physiology specific of ASD [10].

Although several neurotransmitter systems have been investigated in relation to ASD, the hypothesis of serotonin (5-HT) involvement in the neurodevelopment of ASD is one of the longest standing. It was first proposed in 1961 after the discovery of elevated 5-HT levels in blood of a subpopulation of children with ASD [11]. A finding since that time has been shown to be consistent and specific for ASD [12, 13].

In the adult brain, 5-HT is synthesized in serotonergic neurons situated in the raphe nuclei in the brainstem and released throughout the brain, where it has a modulatory effect on 14 different receptor subtypes [14], influencing a wide array of physiological, emotional, and cognitive processes [15]. The primary mechanism decreasing extracellular 5-HT in the adult brain is active transportation by the 5-HT transporter (5-HTT) back into the serotonergic neuron [16]. While 5-HTT function has been shown to also influence blood 5-HT level [17], it is not known how blood 5-HT is related to brain 5-HTT [18], and the mechanism behind the observed hyperserotonemia in ASD is still unknown [19].

In the developing brain, 5-HT has been shown to function both as a neurotransmitter and growth factor, influencing axonal growth, synapse formation, and cortical organization [20, 21]. Supported by preclinical studies, a neurodevelopmental 5-HT hypothesis of ASD has been suggested [19, 22]. But while multiple genetic studies have linked functional variants of the 5-HTT gene SLC6A4 to ASD [19], postmortem studies in ASD show conflicting results [23–25], and in vivo evidence supporting 5-HT involvement in ASD is limited.

While differences in 5-HT synthesis capacity have been reported in children with ASD [26, 27], to date, the 5-HT receptors 2A and 1A and 5-HTT are the only 5-HT markers that have been investigated in vivo in adults with ASD. Murphy et al. found lower 5-HT_2A_ receptor availability in individuals with Asperger’s syndrome using single-photon emission computed tomography (SPECT) [28], but the finding could not be replicated in two successive studies using positron emission tomography (PET) [29, 30]. Using [^18^F]MPPF and PET, Lefevre et al. did not find differences in 5-HT_1A_ receptor availability in a sample of 18 individuals with autism or Asperger’s syndrome [31].

5-HTT was first examined in vivo in ASD by Makkonen et al., who, using SPECT, found regionally lower 5-HTT availability in children and adolescents with autism [32]. This finding was extended to adults by Nakamura et al. who found generalized cortical reductions in 5-HTT availability and a correlation between 5-HTT availability in the cingulate cortex and performance in the Faux Pas test of social cognitive ability in 20 subjects with autism using PET and the radioligand [^11^C]McN-5652 [33]. However, it has been questioned whether [^11^C]McN-5652 is suited for quantifying cortical 5-HTT [34–36]. In an attempt to replicate the earlier findings, Girgis et al. failed to identify significantly lower 5-HTT availability in a sample of eight participants with Asperger’s syndrome using PET and [^11^C]DASB [29].

In the present study, we used PET and the radioligand [^11^C]MADAM to examine 5-HTT availability in a larger sample of adults with ASD and control subjects. [^11^C]MADAM binds with high specificity and selectivity to 5-HTT, and quantification of 5-HTT availability has shown good-to-excellent reliability in both cortical and subcortical regions [37]. All participants were behaviorally phenotyped using comprehensive assessments of social cognition, executive function, and central coherence. In accordance with the neurodevelopmental 5-HT hypothesis of ASD and the findings by Nakamura et al., we hypothesized that 5-HTT availability would be lower in total gray matter in the ASD group, and that 5-HTT availability would be correlated with performance on the Faux Pas test of social cognitive ability, as reported by Nakamura et al. [33]. In addition, we investigated putative associations between 5-HTT availability and behavioral phenotype with an exploratory approach.

## Materials and methods

### Participants

The study was approved by the Regional Ethics Committee in Stockholm County and the Radiation Safety Committee of the Karolinska University Hospital. Verbal and written informed consent were acquired prior to any research activities.

A total of 15 adult participants with ASD (11 men and 4 women) were recruited through tertiary clinics and 15 control participants through local newspapers in the Stockholm region. Participants with ASD had community diagnoses of ASD (ICD-10 or DSM-IV) from specialized units for neurodevelopmental assessments and according to clinical guidelines of Region Stockholm [38], corroborated by the usage of standardized measures, such as the Autism Quotient, Autism Spectrum Diagnostic Interview, and the Ritvo Autism Asperger Diagnostic Scale. In addition, they were assessed using the Autism Diagnostic Observation Schedule-2, module 4. 5-HTT availability has been found to be lower in females, as well as decreasing with age [39, 40]. To allow for paired statistical analysis of PET data, each participant with ASD was matched to a control subject based on sex and age (±3 years). Matching was also done for estimated general intelligence quotient (IQ) (±1 SD), using the matrix-reasoning subtest of Wechsler Adult Intelligence Scale, fourth version. The matrix-reasoning subtest was chosen as an unbiased proxy of WAIS full-scale IQ as it can be regarded ASD fair: nonverbal and less influenced by educational level and social and cultural biases, while highly correlated to WAIS full-scale IQ in a reference population. All subjects underwent psychiatric and somatic screening, including medical history and physical examination, Mini International Neuropsychiatric Interview 6.0b, electrocardiogram, blood and urine tests, and cranial magnetic resonance imaging (MRI). The main exclusion criteria for both groups were (1) the presence of psychiatric disorder according to DSM-IV, (2) IQ < 70, (3) lifetime CNS-related disorder, (4) psychotropic medication within 6 months, (5) nicotine use, (6) substance abuse, (7) pregnancy, and (8) other major health problems. Participants in this study are identical to those described as the Stockholm sample in the previously published article by Horder et al. [41].

### Assessment of behavioral phenotypes

Based on previous research of social and cognitive functioning in ASD, comprehensive behavioral phenotyping was performed using the Reading the Mind in the Eyes test (EYE) [42], the movie for the assessment of social cognition (MASC) [43], and the Faux Pas test [44] for social cognitive performance; verbal fluency subtest letter production (VF lp), category production (VF cp), semantic flexibility (VF sf), the tower test from the Delis–Kaplan executive function system [45], and Conner’s Continuous Performance Test II (CPT) for executive functioning; Embedded Figures Test (EFT) [46] and Fragmented Picture Test (FPT) [47] for central coherence (Supplementary Methods).

### Image acquisition and analysis

[^11^C]MADAM was synthesized as previously described [48] and given as a 10-s intravenous bolus injection followed by a dynamic PET measurement lasting for 93 min. Subjects were examined using an ECAT Exact HR 47 PET system (CTI/Siemens, Knoxville, TN) running in 3D mode with Dual Energy Windows scatter correction, with a resolution of 3.6-mm FWHM at the center of the field of view. PET data were divided into 31 time frames (4 × 15 s, 4 × 30 s, 6 × 60 s, 6 × 180 s, and 11 × 360 s), reconstructed using filtered back projection, and corrected for head motion using a frame-to-first-frame approach. Previously acquired T1-weighted MR images of the brain (3T GE Discovery MR750, GE, Milwaukee, WI) were segmented, and regions of interest (ROIs) were delineated using Freesurfer (version 5.0, http://surfer.nmr.mgh.harvard.edu) [49] according to the atlas of Desikan–Killiany [50]. MR images and ROIs were coregistered to a summated PET image using SPM 5 (Department of Cognitive Neurology, University College London) run in Matlab 2007b for Windows (MATLAB version 7.5, Natick, Massachusetts, The Mathworks Inc.). 5-HTT availability was quantified using the simplified reference tissue model with cerebellum gray matter as the reference region, obtaining binding potentials (BP_ND_) for each ROI [51]. Extraction of time activity curves and parameter estimation was done using in-house developed software running in Matlab (Supplementary Methods). In the primary analysis of 5-HTT availability in total gray matter, a time activity curve was extracted from a mask including all other ROIs in the exploratory analysis, excluding brainstem.

### Statistical analysis

Paired-sample *t* test (two-tailed) was used to investigate differences in demographics, ROI size, tracer administration, and 5-HTT availability in total gray matter between the two groups. The sample size was chosen based on the researchers’ previous experience of clinical PET studies and the results from the two published PET studies on 5-HTT availability in ASD. Expecting the mean effect size reported by Girgis et al. (Cohen’s *d* = 0.78), with *α* = 0.05, a sample of 15 subjects was needed for a power of 0.8 [29].

In an additional analysis, we explored the differences in 5-HTT availability in brainstem and 18 specific subregions within gray matter, group differences in behavioral phenotyping tasks, and associations between regional 5-HTT availability and task performance. The subregions were chosen based on the anatomical and functional properties of the 5-HT system and the putative relevance to ASD. The exploratory analysis included a subgroup analysis of 5-HTT availability in the male and female sample. For comparison with Nakamura et al. [33], an additional exploratory voxel-based analysis was performed post hoc (Supplementary Methods).

Data obtained from PET were assessed for outliers using a conservative application of the Grubb’s test to identify only obvious quantification errors. No subject was considered an outlier in the primary analysis; however, altogether, seven data points were excluded from the exploratory analysis (six in control subjects and one in subjects with ASD). All excluded data points were in the high end of the distribution (Supplementary Methods). For all between-group comparisons, outliers and their matched data point were excluded.

Correlations between 5-HTT availability and behavioral phenotype were explored in the whole data set, under the assumption that ASD represents the extreme end of a continuum of traits rather than a categorical phenotype. While 5-HTT availability data were normally distributed with equal variance, but behavioral assessment data were not, differences in regional 5-HTT availability were investigated using paired-sample *t* test, while differences in behavioral phenotyping tasks and associations between regional 5-HTT availability and task performance were investigated using the nonparametric Wilcoxon signed-rank test and Spearman’s rank-order correlation. For ease of interpretation, results from the exploratory analysis are presented uncorrected for multiple comparisons. However, additional correction for multiple comparisons was done using the method by Benjamini and Hochberg to adjust the false discovery rate to 5% [52]. Based on the degree of interdependency in the data, the number of comparisons was adjusted as described by Cheverud [53] and Nyholt et al. [54] to identify the effective number of independent comparisons (*M*_eff_). Statistical analyses were done using R software (version 3.4.1, R Foundation). The investigator was blinded to subject status during image preprocessing and analysis.

## Results

### Participants

The controls were matched to the subjects with ASD, and the groups did not differ on sex, age, or IQ level; however, a significantly higher educational level was seen in the control group. There was no significant difference in injected or molar activity, and [^11^C]MADAM uptake in the reference region did not differ between the groups (Table 1). The mean ROI volume in the ASD group was on average 2.6% lower (range −6.8 to +4.1%), but only the difference in rostral middle frontal cortex was found to be statistically significant (−6.8%, *p* = 0.03) (Supplementary Table 1).

**Table 1:** Demographic, imaging, and behavioral sample characteristics.

	ASD						Control					
	*n*	Mean	SD	Min		Max	*n*	Mean	SD	Min	Max	*p*
Demographic												
Age	15	33.0	9.1		19.4	48.0	15	33.1	9.4	22.1	49.1	0.92
Education (years)	15	13.7	3.4		9	20	15	16.3	4.0	11	25	0.02
WAIS matrix (sp)^a^	15	12.5	4.0		4	18	15	12.2	3.4	5	17	0.72
Height (cm)	15	177.3	9.6		161	189	15	179.1	8.6	167	194	0.50
Weight (kg)	15	75.8	17.0		50.6	107.7	15	73.7	12.4	51	109	0.59
Imaging												
Injected activity (MBq)	15	366.5	62.6		253	464	15	372.0	55.1	247	440	0.79
Molar activity (GBq/µmol)	15	228.1	85.8		73	403	15	241.3	73.6	106	373	0.71
Injected mass (µg)	15	0.54	0.34		0.24	1.36	15	0.46	0.19	0.20	0.99	0.51
Ref. region AUC (SUV)^b^	15	12.1	1.96		7.8	14.4	15	11.7	1.51	10.0	15.9	0.38
Behavioral												
EYE	15	24.13	5.53		13	30	15	29.60	2.67	24	33	0.005
MASC	15	29.20	5.66		18	37	15	36.13	2.85	28	40	<0.001
Faux Pas	15	43.87	10.51		21	57	15	53.13	5.22	41	60	0.005
VF lp	15	35.13	11.40		10	52	15	49.67	15.15	24	83	0.01
VF cp	15	43.00	10.74		26	69	15	50.67	10.27	31	70	0.06
VF sf	15	12.27	3.22		4	16	15	15.87	2.97	10	20	0.004
Tower	15	519.5	275.9		205	1024	15	453.3	178.5	222	744	0.56
CPT	15	0.60	0.42		0.11	1.87	15	0.87	0.44	0.22	1.8	0.06
EFT	15	726.2	693.9		46	2353	15	468.3	198.4	151	810	0.30
FPT	15	226.3	80.4		131	394	15	213.9	93.4	80	460	0.72

Group mean and standard deviation (SD). Paired-sample *t* test.

*EYE* Reading the Mind in the Eye, *MASC* movie for assessment of social cognition, Faux Pas, *VF lp* verbal fluency letter production, *VF cp* verbal fluency category production, *VF sf* verbal fluency semantic flexibility, *Tower* tower test, *CPT* Conner’s continuous performance test II, *EFT* embedded figure test, *FPT* fragmented picture test.

^a^Scalar points.

^b^Standardized uptake value.

### Performance in behavioral phenotype assessment

For all three social cognitive tests, a significantly lower performance was found in the ASD group, and also a larger variability in performance in the ASD group.

In all tests measuring executive functioning, the ASD group had a lower performance, with statistically significant differences in VF lp and VF cp. The ASD group also exhibited lower performance than controls in tests measuring central coherence. However, in none of these tests, differences between groups reached statistical significance (Table 1).

### 5-HTT availability

**Fig. 1:**
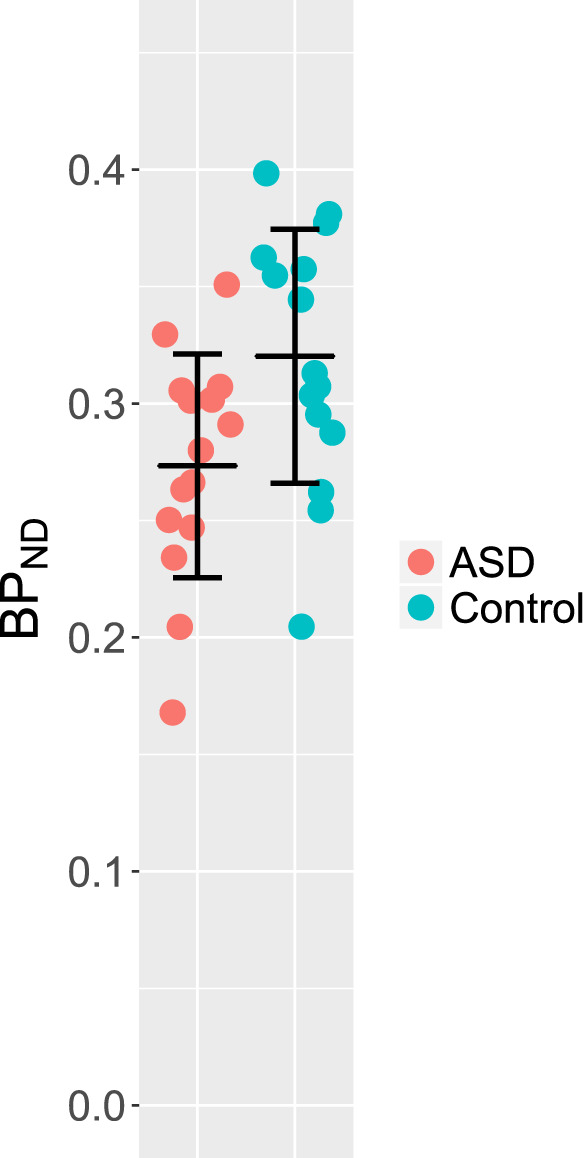
5-HTT availability in total gray matter (BP_ND_). Individual values and group mean. Error bars show ±1 SD. Subjects with ASD and control subjects. ASD BP_ND_ mean [SD] 0.27 [0.05], control BP_ND_ 0.32 [0.05], *p* = 0.004, paired-sample *t* test.

5-HTT availability in total gray matter was found to be 14.6% lower in the ASD group (ASD BP_ND_ mean [SD] 0.27 [0.05], control BP_ND_ 0.32 [0.05]). This difference was statistically significant (*p* = 0.004) (Fig. 1).

**Fig. 2:**
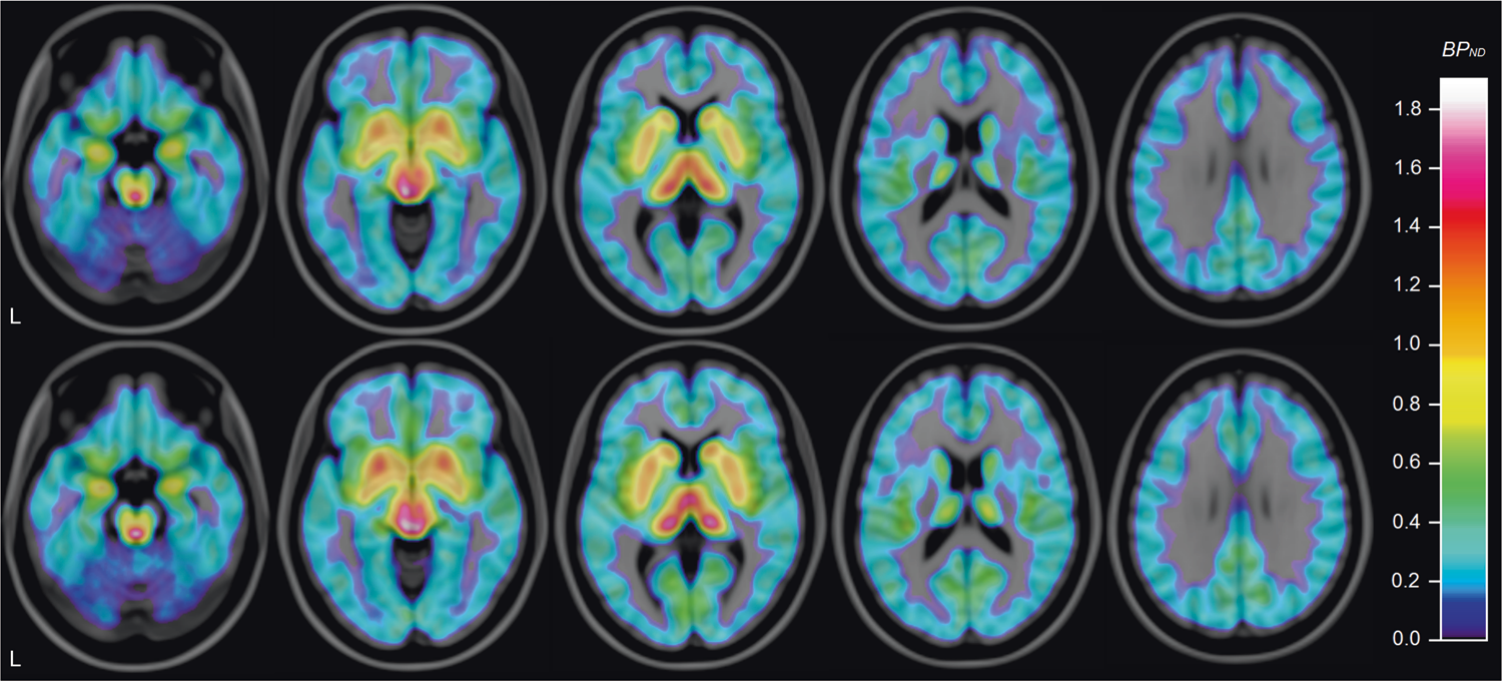
Mean 5-HTT availability in ASD and control group. Average of individual parametric images created using wavelet-aided parametric imaging [89], normalized to MNI-152 template using FSL 5.0 (FMRIB, Oxford, UK) and overlaid on MRI template. Subjects with ASD above, control subjects below. Horizontal slices, MNI coordinates (mm) *z* = −20/−8/4/16/28. “L” indicates left.

In the exploratory analysis, 5-HTT availability was found to be numerically lower in brainstem and all 18 examined subregions of gray matter in the ASD group, with the most marked differences in brainstem, striatal, and cortical areas, where 11–27% lower BP_ND_ was seen. Differences were statistically significant at the *α* = 0.05 level in brainstem and 9 of the 18 subregions: neocortex, frontal cortex, parietal cortex, rostral middle frontal, insular cortex, anterior cingulate cortex, posterior cingulate cortex, nucleus accumbens, and putamen (Table 2, Fig. 2, Supplementary Fig. 1). All findings remained significant after correction for multiple comparisons (*M*_eff_ = 14.04). Subgroup analysis of 5-HTT availability in males and females did not reveal a sex-specific effect (Supplementary Tables 2 and 3). In the additional voxel-based exploratory analysis, two significant clusters were found (Supplementary Fig. 2, Supplementary Table 4).

**Table 2:** Differences in regional 5-HTT availability.

			ASD			Control				
Region of interest			*n*	Mean	SD	*n*	Mean	SD	*p*	Difference
Neocortex			15	0.18	0.04	15	0.22	0.05	0.02*	−16.4%
Frontal cortex			15	0.16	0.04	15	0.20	0.06	0.02*	−19.2%
Occipital cortex			14	0.23	0.06	14	0.26	0.06	0.13	−11.2%
Parietal cortex			15	0.19	0.04	15	0.23	0.05	0.02*	−16.8%
Temporal cortex			15	0.21	0.07	15	0.24	0.06	0.08	−15.7%
Orbitofrontal cortex			13	0.20	0.08	13	0.23	0.10	0.48	−11.4%
Rostral middle frontal cortex			14	0.09	0.04	14	0.12	0.05	0.02*	−27.4%
Fusiform cortex			15	0.27	0.08	15	0.31	0.09	0.08	−13.9%
Insular cortex			15	0.55	0.08	15	0.62	0.11	0.03*	−11.7%
Anterior cingulate cortex			15	0.37	0.06	15	0.46	0.09	0.008*	−19.7%
Posterior cingulate cortex			14	0.30	0.08	14	0.36	0.08	0.02*	−17.1%
Amygdala			14	1.00	0.19	14	1.02	0.18	0.79	−1.8%
Hippocampus			15	0.53	0.13	15	0.57	0.15	0.46	−6.9%
Nucleus accumbens			14	1.03	0.21	14	1.18	0.25	0.03*	−13.1%
Caudate			15	0.63	0.12	15	0.71	0.11	0.12	−11.4%
Putamen			15	1.07	0.15	15	1.23	0.16	0.02*	−12.5%
Pallidum			15	1.20	0.27	15	1.22	0.22	0.80	−1.6%
Thalamus			15	1.05	0.14	15	1.13	0.15	0.10	−7.5%
Brainstem			15	0.73	0.17	15	0.86	0.15	0.008^*^	−15.0%

5-HTT availability (BP_ND_) in 18 subregions of gray matter and brainstem. Group mean and standard deviation (SD). Paired-sample *t* test. Presented numbers are not corrected for multiple comparisons.

*Significant after correction for multiple comparisons.

### Correlations between 5-HTT availability and performance in behavioral phenotype assessment

In the exploratory investigation of associations between regional 5-HTT availability and behavioral phenotype, several significant correlations at the *α* = 0.05 level were found.

Performance in all three social cognitive tests correlated significantly to 5-HTT availability in nucleus accumbens, while performance in two out of three social cognitive tests correlated with 5-HTT availability in anterior cingulate cortex and putamen. Performance in EYE exhibited general correlations to 5-HTT availability in total gray matter as well as in brainstem and 7 of 18 subregions, including frontal cortex, insula, and posterior cingulate cortex.

For tests of executive function, performance in VF lp and VF cp was correlated to 5-HTT availability in nucleus accumbens, while performance in VF sf and CPT correlated significantly to 5-HTT availability in thalamus. Performance in VF sf was also correlated to 5-HTT availability in rostral middle frontal cortex and caudate.

**Fig. 3:**
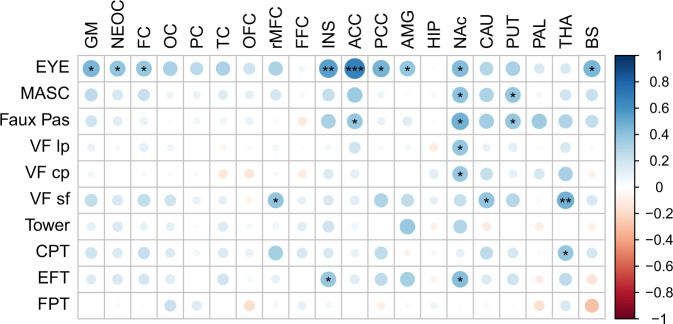
Correlations between 5-HTT availability and performance in behavioral phenotype assessments. Spearman correlation coefficient (*r*_*s*_). GM total gray matter, NEOC neocortex, FC frontal cortex, OC occipital cortex, PC parietal cortex, TC temporal cortex, OFC orbitofrontal cortex, rMFC rostral middle frontal cortex, FFC fusiform cortex, INS insular cortex, ACC anterior cingulate cortex, PCC posterior cingulate cortex, AMG amygdale, HIP hippocampus, NAc nucleus accumbens, CAU caudate, PUT putamen, PAL pallidum, THA thalamus, and BS brainstem. Reading the mind in the eye (EYE), movie for assessment of social cognition (MASC), Faux Pas, verbal fluency letter production (VF lp), verbal fluency category production (VF cp), verbal fluency semantic flexibility (VF sf), tower test (Tower), Conner’s continuous performance test (CPT), embedded figure test (EFT), and fragmented picture test (FPT). In tests Tower, EFT, and FPT, a higher score is indicative of lower performance. For visualization purposes, the sign of *r*_*s*_ for these tests has been changed in the figure to reflect the positive relationship between 5-HTT availability and cognitive performance (*p < 0.05, **p < 0.01, ***p < 0.001, uncorrected for multiple comparisons).

For the tests of central coherence, no significant correlations between performance and 5-HTT availability were found for FPT, while EFT performance correlated significantly to 5-HTT availability in nucleus accumbens and insula (Fig. 3, Supplementary Tables 5 and 6). Of the findings regarding the correlation between regional 5-HTT availability and behavioral phenotype, only the correlation between the social cognitive test EYE and 5-HTT availability in anterior cingulate cortex remained after correction for multiple comparisons (*M*_eff_ = 129.89).

## Discussion

In this study, we investigated the neurodevelopmental 5-HT hypothesis of ASD with the specific aim to replicate and extend the previous finding by Nakamura et al. regarding lower 5-HTT availability in the brain of adults with ASD [33]. In addition, we explored regional 5-HTT availability, and the relationship between 5-HTT availability and behavioral phenotypes of ASD, including a previously reported correlation between 5-HTT availability and performance on the social cognitive test Faux Pas [33]. A selected group of adults with ASD and controls was included to avoid confounding by common comorbidities such as intellectual disability, ADHD, mood and anxiety disorders, and epilepsy.

### Behavioral phenotypes

As expected, the ASD group had a lower performance in all tests examining social cognition, executive function, and central coherence, despite performing similarly on the WAIS matrix-reasoning subtest, here used as an ASD-fair proxy for full-scale IQ. This is consistent with the construct of ASD and confirms the sample as a relevant behavioral phenotype for investigating the neurodevelopmental 5-HT hypothesis of ASD.

### 5-HTT availability

The 5-HTT availability in total gray matter was lower in the ASD group at a statistically significant level, confirming our primary hypothesis of globally lower 5-HTT availability in ASD. Interestingly, while no numerical values of 5-HTT availability were reported by Nakamura et al. [33], the difference in 5-HTT availability in our study was of similar effect size as the nonsignificant results that were reported in the study by Girgis et al. using the radioligand [^11^C]DASB, but including only eight participants with Asperger’s syndrome [29].

The evidence of hyperserotonemia in a subpopulation of individuals with ASD has been well-established for a long time [12]. It has been suggested that high 5-HT levels in the brain during neurodevelopment could inhibit serotonergic neurons through a negative-feedback mechanism mediated by 5-HT_1A_ autoreceptors, causing a permanent loss of 5-HT terminals, and altering the development of targeted brain areas, increasing the likelihood of ASD [22, 55, 56]. This suggestion is supported by animal models of developmental hyperserotonemia exhibiting a reduction of 5-HT terminals, alterations in cortical organization, and dendritic arborization in target tissues, as well as autistic-like behavioral changes [22, 57–59]. However, due to the inherent difficulty quantifying the functional aspects of the human brain in vivo, only two out of 14 known 5-HT receptors have been examined in ASD so far. While these studies provide no or inconclusive evidence of the altered availability of the 5-HT_1A_ and 5-HT_2A_ receptors, these receptor subtypes are known to respond to changes in 5-HT concentration, possibly as a result of externalization/internalization. The unchanged or limited changes in receptor availability found so far in ASD could be the result of a compensatory mechanism.

In this study, we find general decreases in 5-HTT availability, a marker for the 5-HT system, in the cortex, subcortical areas, and in brainstem. Lower 5-HTT availability could in theory reflect a decreased serotonergic innervation with correlated changes in 5-HTT expression, a reduced 5-HTT expression in the presence of unchanged serotonergic innervation, or competition with increased endogenous 5-HT. Since studies suggest that [^11^C]MADAM binding is not sensitive to variability in endogenous 5-HT concentration [60, 61], the difference in 5-HTT availability found is likely to reflect the changes in serotonergic innervation, 5-HTT expression, or both. As the soma and dendrites of serotonergic neurons in the raphe nuclei are the main structures in the brainstem containing 5-HTT, the combination of lower 5-HTT availability in the brainstem and in the cortical and subcortical areas to which the 5-HT axons project, is indicative of functional differences both in somatodendritic and axonal parts of the 5-HT system in ASD. In this aspect, our findings are in line with studies on models of neurodevelopmental hyperserotonemia where reductions in both somatodendritic and axonal development in serotonergic neurons have been reported [21, 62]. Taken together with previous reports on 5-HTT availability in the brains of adult individuals with ASD, our findings corroborate the assumptions drawn from earlier peripheral data in developing and adult autistic individuals having comprehensive alterations in the central 5-HT system.

### Correlations between 5-HTT availability and behavioral phenotype

Although 5-HTT availability might be generally lower in the whole brain of adult subjects with ASD, regional differences may have specific relevance to different aspects of ASD symptoms. The exploratory analysis included a hypothesis-driven investigation of data from both patients and controls, in part replicating and extending the finding of Nakamura et al. in ASD regarding a positive correlation between 5-HTT availability in the anterior cingulate cortex and the social cognitive test of EYE [33]. While associations between 5-HTT availability and multiple behavioral phenotyping tasks were found, tests of social cognition in general and the social cognitive test EYE specifically, demonstrated stronger correlation to 5-HTT availability. Since 5-HTT availability is known to have high interregional correlation, tests with strong correlation to 5-HTT availability in one region would be expected to show correlations to multiple regions. However, due to the design of the social cognitive tests, EYE has been speculated to tap an automated and unconscious, implicit form of social cognition developing in infancy [42, 63], while Faux Pas and MASC have been suggested to test a more deliberate, explicit form of social cognition, emerging later in development with increasing cognitive and linguistic ability [64, 65]. As it has been suggested that ASD is primarily associated with alterations in implicit social cognition [66, 67], the exploratory finding that 5-HTT availability correlates with EYE test performance would be expected for a biomarker of etiological significance.

Performance in all tests of social cognition was correlated to 5-HTT availability in the nucleus accumbens. Interestingly, increased 5-HT signaling in nucleus accumbens has rescued social deficits in a preclinical model of autism [68], and while coordinated signaling of 5-HT and oxytocin in the nucleus accumbens has been reported to be required for the reinforcement of social interaction [69], investigations of oxytocin and 5-HT indicate a different pattern of interaction in subjects with ASD compared with controls [31, 70–72].

### 5-HT hypothesis of ASD

Since hyperserotonemia has been reported only in approximately one-third of individuals with ASD, this does not account for the etiology in the majority of individuals with ASD [12]. Also, hyperserotonemia has been shown in first-degree relatives not diagnosed with ASD [73–75]. Because 5-HT does not cross the mature blood–brain barrier, it has been proposed that a maternal hyperserotonemia could influence fetal neurodevelopment, while not necessarily contributing to ASD symptoms in the adult [20, 76]. However, preclinical studies indicate no impact of maternal hyperserotonemia on fetal 5-HT levels in the forebrain, but instead suggest increased placental output of 5-HT linked to maternal genetic hyperserotonemia [20, 56, 77]. In addition, a number of environmental factors have been suggested to transiently influence serotonergic tone in the brain during fetal development, factors that would not be persistent or directly detectable in the adult. It has been suggested that inflammatory responses or vitamin D deficiencies of the mother could increase the output of 5-HT from the placenta, and increase the risk of ASD [78–80]. In utero exposure to valproic acid has been linked to ASD, and has been shown to increase 5-HT levels and inhibit the development of serotonergic neurons in preclinical models [81–84]. While investigations in preclinical models of maternal stress, depression, and treatment with selective 5-HT-reuptake inhibitors (SSRIs) indicate a possible significant impact on the 5-HT system, and are linked to altered neurological and behavioral development in offspring [56], epidemiological studies have not been able to link the use of SSRIs during pregnancy to increased incidence of ASD [85, 86]. Although inconclusive, the investigations of the 5-HT system in ASD suggest that an interaction between multiple genetic and environmental factors could alter the serotonergic tone in the brain during key stages of 5-HT system maturation, shifting the neurodevelopmental trajectory toward an ASD phenotype.

### Limitations

In clinical PET studies, systematic differences in nonspecific binding between groups may introduce a bias in estimated target availability. In this study, the AUC of standardized uptake of radioactivity in the reference region was used as a proxy for nonspecific binding to investigate differences between groups. Without arterial blood data, it is not possible to determine if the 3.1% difference in the reference region AUC is attributable to true differences in nonspecific binding introducing bias, or peripheral sources. Notably, this difference is small, did not reach statistical significance, and is of opposite direction as found by Nakamura et al. where arterial blood sampling was used [33]. As such, it should be regarded as an expected variation in imaging characteristics and not an indication of bias.

Systematical differences in ROI volume may also produce biased estimates in target availability driven by partial volume effects. Although ROI volumes were numerically smaller in the ASD group, this difference did not reach statistical significance for any ROI but the rostral middle frontal region. While partial volume effect correction can reduce bias caused by volumetric differences, it also introduces noise to the data. Since the average differences in ROI volumes were small and generally below 5%, data were not corrected for partial volume effects. Thus, it cannot be excluded that the differences in 5-HTT availability in some regions (notably rostral middle frontal region) may have been emphasized by a limited partial volume effect.

In the exploratory analysis, 5-HTT availability was examined in multiple regions, and correlations to multiple behavioral phenotyping tasks were tested. This increases the risk of type-1 errors, and while the results are presented uncorrected for ease of interpretation, the correlational data on their own should be interpreted with caution. Correcting for multiple comparisons in the correlation analysis, only the correlation between the social cognitive test EYE and 5-HTT availability in the anterior cingulate cortex reached the significance threshold. However, the fact that we replicate previous findings regarding statistically significant differences in 5-HTT availability, and in part also correlations between specific behavioral phenotype assessments and regional 5-HTT availability, is in itself indicative of a true finding. While a selected, well-matched ASD sample decreases the risk of confounding, our results are not necessarily generalizable to individuals with ASD and intellectual disability, this is at the same time one of the main strengths of the study as it allows for the examination of an ASD-specific phenotype.

## Conclusions

Here, we confirm our primary hypothesis that 5-HTT availability is significantly lower in total gray matter of adults with ASD compared with control subjects. An exploratory analysis also supports lower availability in the brainstem. We find correlations between 5-HTT availability and several behavioral phenotyping tasks relevant for ASD. Specifically, we replicate and extend the previous finding of a correlation between 5-HTT availability in the anterior cingulate cortex and performance in social cognition. Our findings extend previous PET studies endorsing the central role of 5-HT in the physiology of ASD. The lower 5-HTT availability we report in adult ASD is consistent with the hypothesis of a hyperserotonergic tone in neurodevelopment toward an ASD phenotype. The 5-HTT can be seen as a marker for the 5-HT system, but is primarily a mechanism regulating extracellular 5-HT in the brain, making it a possible target for pharmacological interventions in ASD. It is not possible to determine whether serotonergic tone is higher, lower, or unchanged in adult ASD based on current knowledge, and pharmacological interventions blocking 5-HTT in ASD using selective 5-HT-reuptake inhibitors have not shown effectiveness. However, evidence is limited [87]. While 5-HT exerts its function through 14 different receptors, only the 5-HT_2A_ and 5-HT_1A_ receptor has been examined in vivo in relation to ASD. Based on its localization on presynaptic terminals, role in 5-HT release, influence on 5-HTT function [88], and suggested role in social reward [69], the 5-HT_1B_ receptor would be an interesting target for future studies. Continued efforts to understand the role of 5-HT in the neurodevelopment and ASD by investigating different aspects of the 5-HT system in clinical studies as well as in preclinical models are warranted.

## Supplementary information

Supplemental Material
