# Supplementary material for: Serotonin transporter availability in adults with autism—a positron emission tomography study
Source: Mol Psychiatry. 2020 Aug 26;26(5):1647–58. doi: 10.1038/s41380-020-00868-3 (PMC8159737; doi:10.1038/s41380-020-00868-3)
Supplement: Supplementary file 1 — Supplemental Material [file 41380_2020_868_MOESM1_ESM.docx]

**Supplementary material**

1. **Supplementary methods**
2. **Supplementary Figure 1** - Regional 5-HTT availability (BP_ND_)
3. **Supplementary Figure 2** - Illustration of results from voxel analysis
4. **Supplementary Table 1** - ROI size
5. **Supplementary Table 2** - Regional 5-HTT availability (BP_ND_) in male subjects.
6. **Supplementary Table 3** - Regional 5-HTT availability (BP_ND_) in female subjects.
7. **Supplementary Table 4** - Results from Voxel-wise analysis.
8. **Supplementary Table 5** - Correlations between 5-HTT availability and performance in behavioral phenotype assessments. Spearman correlations coefficients(*r_s_*).
9. **Supplementary Table 6** - Correlations between 5-HTT availability and performance in behavioral phenotype assessments. Spearman correlations (*p*).

**Supplementary methods**

***Assessment of behavioral phenotypes***

Social cognition abilities including theory of mind were assessed with Reading the mind in the eyes (EYE), Movie for assessment of social cognition (MASC) and Faux pas test. In the EYE test, the participant identifies the emotion expressed by each eye pair. MASC is a computerized test showing sequences of social situations with questions to be answered. In Faux pas, the participant is presented with stories of social situations and should detect social rule violations. Executive functioning was measured with Verbal fluency and Tower Test from the Delis-Kaplan Executive Function System (D-KEFS) and Conner’s Continuous Performance Test II(CPT-II). The Verbal fluency test consists of three subtests measuring letter production, category production and semantic flexibility, where the participant should produce as many words as possible according to instructions. Tower of Hanoi is a measure of planning and problem solving, where the participant is required to rearrange a set of disks according to instructions. The computerized test of CPT-II evaluates sustained attention, vigilance and inhibition. The participant is asked to respond to different stimuli as fast as possible by pressing a button on the computer. Central coherence was evaluated with Embedded Figure Test (EFT) and Fragmented Picture Test (FPT). In EFT, the subject is instructed to detect a target embedded within a complex background. In FPT, the participant is shown a series of pictures, from most fragmented to complete, and asked to identify what the picture represent.

***Image acquisition and analysis***

Prior to examination an individually fitted plaster helmet was constructed to minimize head movement during examination. A 10 min transmission scan with three rotating ^68^Ge-rods was conducted before image acquisition to correct for attenuation and scatter. Cerebellum grey matter is thought to lack specific binding of [^11^C]MADAM, and to prevent spill in to reference region from regions containing specific 5-HTT binding (vermis, nucleus dentate and cerebral cortex) an eroded ROI of cerebellum grey matter was used as reference region. For one control subject, the automated delineation of cerebellum could not be used. When investigating correlations between [^11^C]MADAM binding and cognitive performance this subject was excluded. For all other analyzes, cerebellum grey matter for this subject and its matched case, was manually delineated by an investigator blinded to subject status. Voxels at the limit of field of view of PET were excluded from the analysis in a uniform way for all subjects due to increase in noise and to secure compatible measurements. Since volumetric differences of cortical areas in ASD has previously been found, and group differences in cortical volume can lead to apparent differences in BP_ND_ due to partial volume effects, an investigation of differences in ROI size between groups was done.

An additional voxel-based analysis was carried out post hoc for hypothesis free exploration of differences in 5-HTT availability. Individual parametric images were created using 3D stationary wavelet aided parametric imaging and non-invasive Logan with multilinear fitting.[1] The individual parametric PET images were resliced to MR-space and normalized to MNI-152 template (2mm voxels) using warping parameters obtain by a sequential employment of BET, FLIRT and FNIRT in FSL 5.0 (FMRIB, Oxford, UK) as described in detail by Matheson et al.[2] The same warping parameters and the same procedure were applied to the individual grey matter masks used for the total grey matter mask, with the addition of brainstem. In FSL the warped parametric images and masks were smoothed using an 8mm FWHM 3D gaussian kernel. The individual smoothed grey matter masks were averaged and thresholded at 30% to create a binary mask of relevant voxels.

***Statistical analysis***

Differences in variances between groups were assessed using F-test. In small regions or regions with low 5-HTT availability there is a low signal to noise ratio. This can sometimes result in biologically impossible or extremely unlikely results. To identify possible outliers due to quantification error the Grubb’s test was applied on the data for each ROI. No single subject was identified as an outlier as a whole. Ten datapoints, all in the high end, six of which had a G-score >3 were identified as significant outliers. Since it is known that 5-HTT availability show a high degree of correlation across regions within a subject, in a second step, we used a dataset in which 5-HTT availability had been normalized across subjects to the individual total grey matter 5-HTT availability. This was done to make sure not to exclude unusual, but biologically possible extreme values in subjects with generally extreme 5-HTT availability. The six data points with a G-score >3 in the raw data, all were identified as outliers also in the normalized data and excluded (5 in control group, 1 in ASD group). Of the remaining four data points identified as outliers in the raw data, two were not identified as outliers in the normalized data and were not excluded. The remaining two data points with G < 3 were individually assessed, resulting in the exclusion of the data point with the higher G-score (control subject). All together seven data points were excluded from the analysis (6 in control subjects, 1 in subject with ASD). All excluded data points were in the high end of the distribution.

A voxel based paired T-test was performed in SPM12 (Department of Cognitive Neurology, University College London) running in Matlab 2014b (MATLAB version 8.4.0, Natick, Massachusetts; The Mathworks Inc.), limiting the investigation to the positive value voxels in the previously created grey matter/brainstem mask. The initial threshold was set to α = 0.05, family-wise error corrected, with a cluster extend threshold of 20 voxels.

***Participants***

While partial volume effects correction can reduce bias caused by volumetric differences, it also introduces noise to the data. Since average differences in cortical volume were small and generally below 5%, data was not corrected for partial volume effects.

***Results***

The voxel-based analysis revealed two clusters, one in the left posterior cingulate cortex and one in left middle frontal cortex, with significantly lower binding in the ASD group. (Supplementary Figure 2).

**
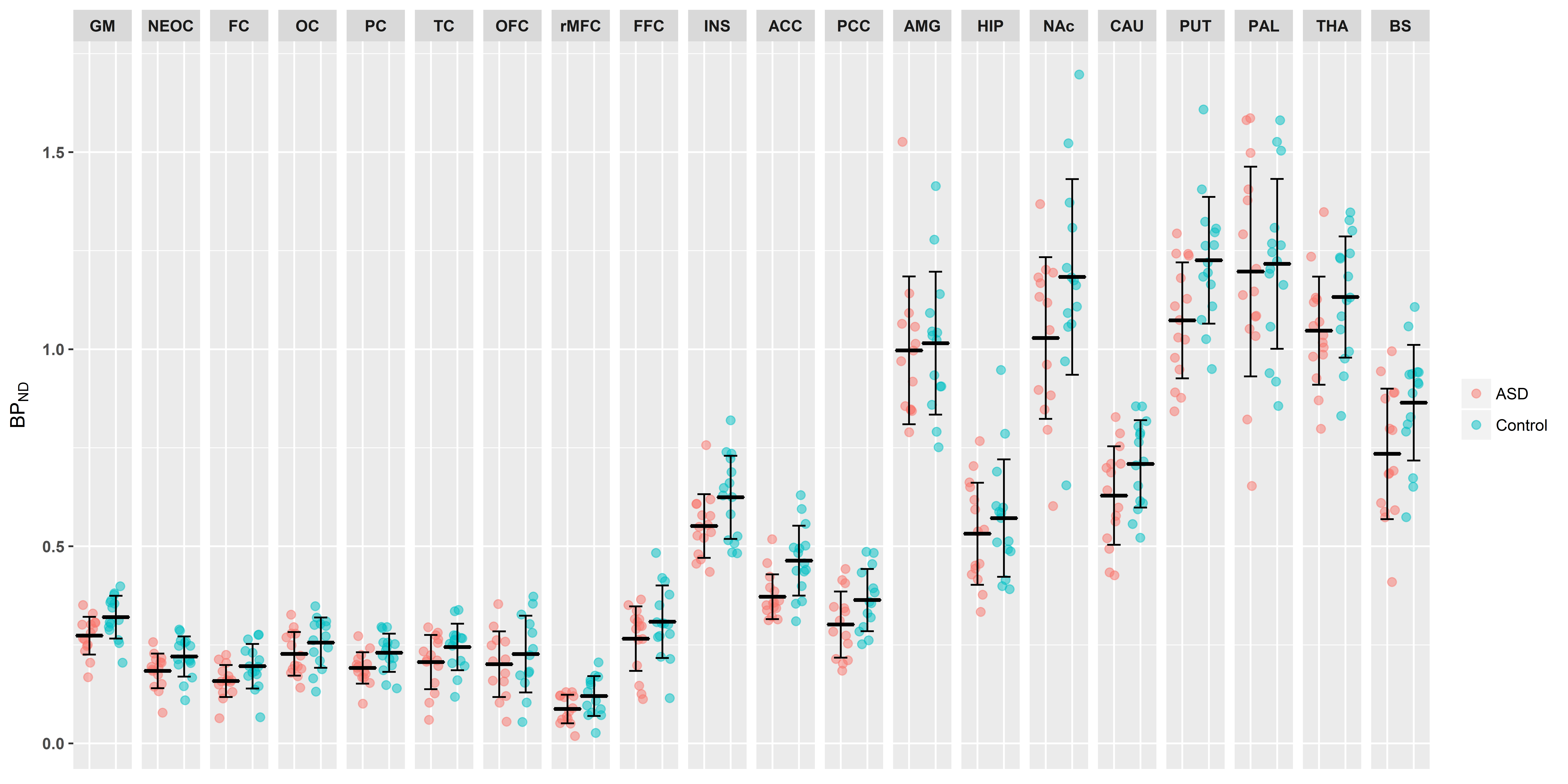
Supplementary Figure 1. Regional 5-HTT availability**

Supplementary Figure 1. Regional 5-HTT availability (BP_ND_). Individual values and group mean. Error bars show ± 1 SD. Autism spectrum disorder (ASD) and control subjects.

**Supplementary Figure 2. Illustration of results from voxel analysis**

**
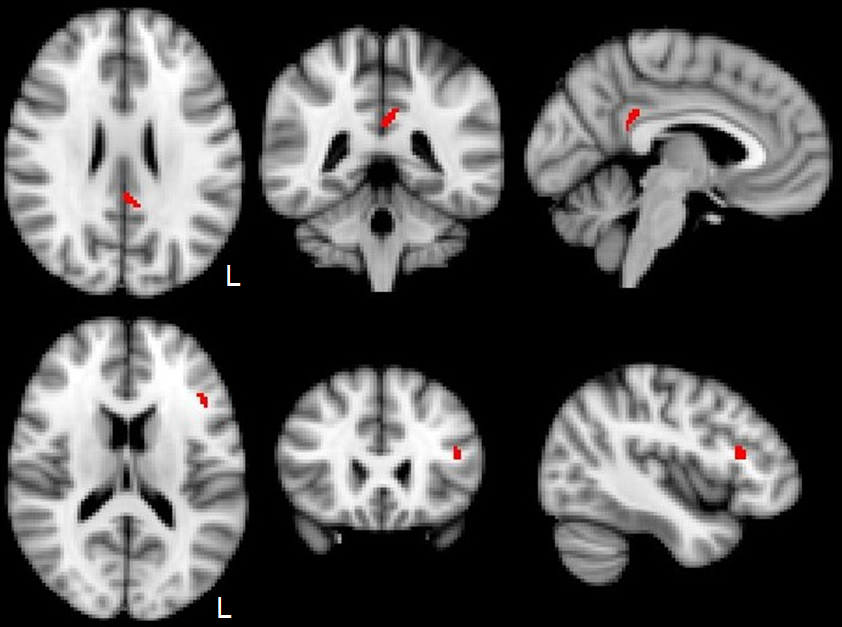
**

Figure 2. Results from voxel analysis. Clusters with significantly lower 5-HTT availability in ASD group. Cluster extent overlaid on MNI-152 template. Left posterior cingulate cortex and left middle frontal cortex. “L” indicate left.

**Supplementary Table 1. ROI size**

|  |  |  | **ASD** |  | **Control** |  |  |  |
| --- | --- | --- | --- | --- | --- | --- | --- | --- |
| **Region of interest** | | | mean | SD | mean | SD | *p* | Difference |
| Total grey matter | | | 4.87E+05 | 4.81E+04 | 5.02E+05 | 5.08E+04 | 0.19 | -3.0% |
| Neocortex | | | 3.89E+05 | 4.05E+04 | 4.02E+05 | 4.19E+04 | 0.19 | -3.2% |
| Frontal cortex | | | 1.45E+05 | 1.69E+04 | 1.51E+05 | 1.77E+04 | 0.14 | -4.3% |
| Occipital cortex | | | 4.75E+04 | 6.34E+03 | 4.86E+04 | 5.97E+03 | 0.51 | -2.3% |
| Parietal cortex | | | 9.23E+04 | 1.02E+04 | 9.62E+04 | 1.28E+04 | 0.25 | -4.1% |
| Temporal cortex | | | 1.08E+05 | 9.40E+03 | 1.09E+05 | 1.13E+04 | 0.74 | -0.7% |
| Orbitofrontal cortex | | | 2.25E+04 | 2.98E+03 | 2.36E+04 | 2.45E+03 | 0.16 | -4.7% |
| Rostral middle frontal cx | | | 3.53E+04 | 4.20E+03 | 3.79E+04 | 4.58E+03 | 0.03 | -6.8% |
| Fusiform cortex | | | 2.14E+04 | 3.06E+03 | 2.15E+04 | 3.24E+03 | 0.93 | -0.3% |
| Insular cortex | | | 1.45E+04 | 1.12E+03 | 1.49E+04 | 1.14E+03 | 0.25 | -2.7% |
| Anteror cingulate cortex | | | 1.00E+04 | 2.18E+03 | 1.00E+04 | 2.27E+03 | 0.99 | 0.1% |
| Posterior cingulate cortex | | | 1.20E+04 | 1.77E+03 | 1.23E+04 | 1.70E+03 | 0.65 | -1.7% |
| Amygdala | | | 3.60E+03 | 5.89E+02 | 3.78E+03 | 4.98E+02 | 0.30 | -4.9% |
| Hippocampus | | | 8.97E+03 | 1.07E+03 | 9.30E+03 | 9.98E+02 | 0.22 | -3.5% |
| Nucleus accumbens | | | 1.33E+03 | 1.79E+02 | 1.27E+03 | 1.63E+02 | 0.31 | 4.1% |
| Caudate | | | 8.02E+03 | 1.26E+03 | 8.13E+03 | 1.19E+03 | 0.72 | -1.4% |
| Putamen | | | 1.12E+04 | 1.07E+03 | 1.18E+04 | 1.30E+03 | 0.08 | -5.1% |
| Pallidum | | | 3.39E+03 | 5.21E+02 | 3.48E+03 | 4.74E+02 | 0.60 | -2.6% |
| Thalamus | | | 1.57E+04 | 1.82E+03 | 1.61E+04 | 1.54E+03 | 0.44 | -2.5% |
| Brainstem | | | 2.00E+04 | 2.52E+03 | 2.06E+04 | 2.62E+03 | 0.43 | -2.6% |

Table 1. Size of ROIs, total grey matter, 18 subregions of grey matter and brainstem. Group mean and standard deviation (SD).

**Supplementary Table 2. Regional 5-HTT availability (BP_ND_) in male subjects**

|  |  |  | **ASD** |  |  | **Control** |  |  |  |  |
| --- | --- | --- | --- | --- | --- | --- | --- | --- | --- | --- |
| **Region of interest** | | | n | mean | SD | n | mean | SD | *p* | Difference |
| Total grey matter | | | 11 | 0.27 | 0.05 | 11 | 0.32 | 0.06 | 0.005 | -16.2% |
| Neocortex | | | 11 | 0.18 | 0.05 | 11 | 0.22 | 0.05 | 0.03 | -18.4% |
| Frontal cortex | | | 11 | 0.16 | 0.05 | 11 | 0.19 | 0.06 | 0.07 | -18.6% |
| Occipital cortex | | | 10 | 0.21 | 0.06 | 10 | 0.25 | 0.07 | 0.17 | -14.1% |
| Parietal cortex | | | 11 | 0.19 | 0.04 | 11 | 0.23 | 0.04 | 0.03 | -18.5% |
| Temporal cortex | | | 11 | 0.19 | 0.08 | 11 | 0.24 | 0.06 | 0.07 | -20.2% |
| Orbitofrontal cortex | | | 10 | 0.20 | 0.09 | 10 | 0.22 | 0.10 | 0.56 | -11.7% |
| Rostral middle frontal cx | | | 10 | 0.08 | 0.04 | 10 | 0.12 | 0.05 | 0.05 | -26.9% |
| Fusiform cortex | | | 11 | 0.25 | 0.09 | 11 | 0.30 | 0.10 | 0.14 | -16.1% |
| Insular cortex | | | 11 | 0.56 | 0.09 | 11 | 0.62 | 0.10 | 0.09 | -9.9% |
| Anteror cingulate cortex | | | 11 | 0.38 | 0.06 | 11 | 0.48 | 0.09 | 0.02 | -20.6% |
| Posterior cingulate cortex | | | 10 | 0.30 | 0.08 | 10 | 0.37 | 0.09 | 0.02 | -20.0% |
| Amygdala | | | 10 | 0.95 | 0.11 | 10 | 0.98 | 0.15 | 0.68 | -2.6% |
| Hippocampus | | | 11 | 0.52 | 0.12 | 11 | 0.57 | 0.16 | 0.40 | -9.5% |
| Nucleus accumbens | | | 10 | 1.06 | 0.21 | 10 | 1.17 | 0.23 | 0.05 | -9.8% |
| Caudate | | | 11 | 0.66 | 0.12 | 11 | 0.72 | 0.11 | 0.37 | -7.5% |
| Putamen | | | 11 | 1.07 | 0.15 | 11 | 1.21 | 0.12 | 0.009 | -11.6% |
| Pallidum | | | 11 | 1.19 | 0.26 | 11 | 1.27 | 0.20 | 0.38 | -6.5% |
| Thalamus | | | 11 | 1.08 | 0.13 | 11 | 1.14 | 0.16 | 0.33 | -5.0% |
| Brainstem | | | 11 | 0.71 | 0.16 | 11 | 0.84 | 0.16 | 0.02 | -15.5% |

Table 2. 5-HTT availability (BP_ND_) in total grey matter, 18 subregions of grey matter and brainstem. Group mean and standard deviation (SD).

**Supplementary Table 3. Regional 5-HTT availability (BP_ND_) in female subjects**

|  |  |  | **ASD** |  |  | **Control** |  |  |  |  |
| --- | --- | --- | --- | --- | --- | --- | --- | --- | --- | --- |
| **Region of interest** | | | n | mean | SD | n | mean | SD | *p* | Difference |
| Total grey matter | | | 4 | 0.29 | 0.02 | 4 | 0.32 | 0.06 | 0.41 | -10.4% |
| Neocortex | | | 4 | 0.20 | 0.01 | 4 | 0.23 | 0.05 | 0.42 | -11.3% |
| Frontal cortex | | | 4 | 0.16 | 0.01 | 4 | 0.20 | 0.06 | 0.23 | -20.7% |
| Occipital cortex | | | 4 | 0.26 | 0.05 | 4 | 0.27 | 0.04 | 0.54 | -4.4% |
| Parietal cortex | | | 4 | 0.20 | 0.04 | 4 | 0.23 | 0.07 | 0.46 | -12.3% |
| Temporal cortex | | | 4 | 0.24 | 0.02 | 4 | 0.25 | 0.06 | 0.79 | -3.8% |
| Orbitofrontal cortex | | | 3 | 0.22 | 0.04 | 3 | 0.24 | 0.11 | 0.76 | -10.4% |
| Rostral middle frontal cx | | | 4 | 0.09 | 0.03 | 4 | 0.13 | 0.06 | 0.27 | -28.3% |
| Fusiform cortex | | | 4 | 0.32 | 0.03 | 4 | 0.35 | 0.06 | 0.42 | -8.8% |
| Insular cortex | | | 4 | 0.53 | 0.06 | 4 | 0.63 | 0.13 | 0.25 | -16.4% |
| Anteror cingulate cortex | | | 4 | 0.35 | 0.03 | 4 | 0.42 | 0.09 | 0.28 | -17.0% |
| Posterior cingulate cortex | | | 4 | 0.31 | 0.10 | 4 | 0.34 | 0.05 | 0.60 | -9.2% |
| Amygdala | | | 4 | 1.11 | 0.31 | 4 | 1.11 | 0.23 | 1.00 | -0.1% |
| Hippocampus | | | 4 | 0.57 | 0.15 | 4 | 0.57 | 0.13 | 1.00 | 0.1% |
| Nucleus accumbens | | | 4 | 0.95 | 0.18 | 4 | 1.21 | 0.33 | 0.29 | -21.1% |
| Caudate | | | 4 | 0.54 | 0.12 | 4 | 0.69 | 0.13 | 0.28 | -22.2% |
| Putamen | | | 4 | 1.08 | 0.17 | 4 | 1.27 | 0.26 | 0.41 | -14.8% |
| Pallidum | | | 4 | 1.22 | 0.33 | 4 | 1.06 | 0.19 | 0.23 | 14.6% |
| Thalamus | | | 4 | 0.95 | 0.12 | 4 | 1.11 | 0.17 | 0.16 | -14.6% |
| Brainstem | | | 4 | 0.80 | 0.18 | 4 | 0.93 | 0.11 | 0.33 | -13.9% |

Table 3. 5-HTT availability (BP_ND_) in total grey matter, 18 subregions of grey matter and brainstem. Group mean and standard deviation (SD).

**Supplementary Table 4. Results voxel analysis**

| cluster-level | | | | peak-level | | | | |  |
| --- | --- | --- | --- | --- | --- | --- | --- | --- | --- |
| *p*_FWE-corr_ | *q*_FDR-corr_ | *k*_E_ | *p*_uncorr_ | *p*_FWE-corr_ | *q*_FDR-corr_ | *T* | *Z* | *p*_uncorr_ | mm |
| 0.023 | 0.898 | 41 | 0.449 | 0.011 | 0.613 | 6.69 | 4.41 | 0.000 | -4,-42,26 |
| 0.030 | 0.898 | 21 | 0.599 | 0.023 | 0.613 | 6.11 | 4.20 | 0.000 | -42,24,16 |

**Supplementary Table 5. Correlations between 5-HTT availability and performance in behavioral phenotype assessments. Spearman correlations coefficients(*r_s_*).**

|  | GM | NEOC | FC | OC | PC | TC | OFC | rMFC | FFC | INS | ACC | PCC | AMG | HIP | NAc | CAU | PUT | PAL | THA | BS |
| --- | --- | --- | --- | --- | --- | --- | --- | --- | --- | --- | --- | --- | --- | --- | --- | --- | --- | --- | --- | --- |
| EYE | 0.46 | 0.39 | 0.39 | 0.33 | 0.26 | 0.31 | 0.21 | 0.31 | 0.06 | 0.55 | 0.68 | 0.47 | 0.38 | 0.02 | 0.44 | 0.29 | 0.33 | 0.16 | 0.16 | 0.45 |
| MASC | 0.25 | 0.17 | 0.22 | 0.07 | 0.08 | 0.14 | 0.11 | 0.18 | -0.04 | 0.24 | 0.36 | 0.07 | 0.02 | -0.04 | 0.40 | 0.32 | 0.38 | 0.05 | 0.19 | 0.22 |
| Faux Pas | 0.20 | 0.13 | 0.10 | -0.02 | 0.06 | 0.06 | 0.09 | -0.03 | -0.12 | 0.33 | 0.38 | 0.10 | 0.13 | -0.01 | 0.48 | 0.34 | 0.38 | 0.36 | 0.31 | 0.25 |
| VF lp | 0.09 | 0.03 | 0.12 | 0.07 | 0.01 | -0.05 | -0.04 | 0.06 | -0.02 | 0.05 | 0.20 | -0.04 | 0.06 | -0.10 | 0.39 | 0.09 | 0.12 | -0.03 | 0.17 | 0.03 |
| VF cp | 0.06 | 0.01 | 0.03 | 0.03 | 0.02 | -0.12 | -0.14 | 0.04 | -0.11 | 0.15 | 0.12 | -0.01 | 0.00 | 0.13 | 0.38 | 0.24 | 0.10 | 0.17 | 0.32 | -0.07 |
| VF sf | 0.25 | 0.17 | 0.24 | 0.21 | 0.09 | 0.11 | -0.06 | 0.39 | 0.10 | 0.16 | 0.13 | 0.30 | 0.25 | 0.10 | 0.23 | 0.40 | 0.29 | 0.04 | 0.48 | 0.17 |
| Tower | 0.11 | 0.16 | 0.10 | 0.11 | -0.03 | 0.15 | 0.05 | 0.07 | 0.13 | 0.21 | 0.07 | 0.01 | 0.37 | -0.07 | 0.29 | -0.11 | -0.01 | -0.10 | 0.01 | -0.07 |
| CPT | 0.20 | 0.16 | 0.22 | 0.14 | 0.06 | 0.16 | 0.10 | 0.34 | 0.17 | 0.16 | 0.09 | 0.26 | -0.06 | -0.04 | 0.10 | 0.26 | 0.17 | -0.02 | 0.38 | 0.17 |
| EFT | 0.14 | 0.16 | 0.18 | 0.14 | 0.00 | 0.20 | 0.04 | 0.10 | 0.01 | 0.38 | 0.14 | 0.25 | 0.34 | -0.06 | 0.42 | 0.15 | 0.21 | -0.09 | 0.25 | -0.15 |
| FPT | 0.01 | 0.04 | 0.02 | 0.21 | 0.15 | 0.01 | -0.18 | 0.04 | 0.11 | 0.06 | 0.02 | -0.09 | 0.04 | 0.07 | -0.01 | -0.04 | 0.02 | -0.16 | 0.16 | -0.30 |

Table 5. Total grey matter (GM), neocortex (NEOC), frontal cortex (FC), occipital cortex (OC), parietal cortex (PC), temporal cortex (TC), orbitofrontal cortex (OFC), rostral middle frontal cortex (rMFC), fusiform cortex (FFC), insular cortex (INS), anterior cingulate cortex (ACC), posterior cingulate cortex (PCC), amygdale (AMG), hippocampus (HIP), nucleus accumbens (NAc), caudate (CAU), putamen (PUT), pallidum (PAL), thalamus (THA), brainstem (BS). Reading the mind in the eye (EYE), Movie for assessment of social cognition (MASC), Faux pas, Verbal fluency letter production (VF lp), Verbal fluency category production (VF cp), Verbal fluency semantic flexibility (VF sf), Tower test (Tower), Conner’s continuous performance test (CPT), Embedded figure test (EFT), Fragmented picture test (FPT). In tests Tower, EFT and FPT, a higher score is indicative of lower performance. For visualisation purposes the sign of *r_s_* for these tests has been changed in the figure to reflect the positive relationship between 5-HTT availability and cognitive performance.

**Supplementary Table 6. Correlations between 5-HTT availability and performance in behavioral phenotype assessments. Spearman correlations (*p*).**

|  | GM | NEOC | FC | OC | PC | TC | OFC | rMFC | FFC | INS | ACC | PCC | AMG | HIP | NAc | CAU | PUT | PAL | THA | BS |
| --- | --- | --- | --- | --- | --- | --- | --- | --- | --- | --- | --- | --- | --- | --- | --- | --- | --- | --- | --- | --- |
| EYE | 0.013 | 0.035 | 0.039 | 0.091 | 0.167 | 0.096 | 0.299 | 0.104 | 0.773 | 0.002 | 0.000 | 0.012 | 0.046 | 0.922 | 0.019 | 0.133 | 0.084 | 0.406 | 0.393 | 0.013 |
| MASC | 0.182 | 0.365 | 0.246 | 0.721 | 0.665 | 0.467 | 0.572 | 0.354 | 0.835 | 0.216 | 0.052 | 0.713 | 0.914 | 0.832 | 0.034 | 0.095 | 0.040 | 0.790 | 0.311 | 0.244 |
| Faux Pas | 0.288 | 0.502 | 0.621 | 0.927 | 0.760 | 0.743 | 0.660 | 0.875 | 0.541 | 0.082 | 0.040 | 0.626 | 0.501 | 0.945 | 0.010 | 0.067 | 0.041 | 0.056 | 0.106 | 0.191 |
| VF lp | 0.649 | 0.871 | 0.548 | 0.741 | 0.976 | 0.784 | 0.851 | 0.768 | 0.931 | 0.785 | 0.286 | 0.859 | 0.777 | 0.600 | 0.041 | 0.632 | 0.537 | 0.893 | 0.388 | 0.875 |
| VF cp | 0.768 | 0.964 | 0.873 | 0.893 | 0.910 | 0.518 | 0.500 | 0.841 | 0.579 | 0.430 | 0.538 | 0.955 | 0.988 | 0.508 | 0.047 | 0.213 | 0.589 | 0.373 | 0.089 | 0.723 |
| VF sf | 0.194 | 0.372 | 0.200 | 0.290 | 0.634 | 0.571 | 0.778 | 0.039 | 0.593 | 0.397 | 0.505 | 0.116 | 0.196 | 0.603 | 0.230 | 0.032 | 0.130 | 0.854 | 0.009 | 0.386 |
| Tower | 0.578 | 0.414 | 0.620 | 0.593 | 0.895 | 0.440 | 0.802 | 0.740 | 0.518 | 0.285 | 0.728 | 0.947 | 0.051 | 0.701 | 0.134 | 0.553 | 0.978 | 0.620 | 0.960 | 0.701 |
| CPT | 0.293 | 0.421 | 0.247 | 0.468 | 0.759 | 0.419 | 0.634 | 0.081 | 0.378 | 0.404 | 0.636 | 0.184 | 0.744 | 0.840 | 0.595 | 0.181 | 0.365 | 0.898 | 0.041 | 0.367 |
| EFT | 0.458 | 0.399 | 0.341 | 0.481 | 0.991 | 0.301 | 0.825 | 0.597 | 0.943 | 0.043 | 0.477 | 0.195 | 0.077 | 0.744 | 0.025 | 0.427 | 0.279 | 0.628 | 0.185 | 0.440 |
| FPT | 0.977 | 0.851 | 0.922 | 0.280 | 0.451 | 0.979 | 0.380 | 0.824 | 0.587 | 0.770 | 0.926 | 0.650 | 0.858 | 0.714 | 0.950 | 0.846 | 0.928 | 0.405 | 0.404 | 0.119 |

Table 6. Total grey matter (GM), neocortex (NEOC), frontal cortex (FC), occipital cortex (OC), parietal cortex (PC), temporal cortex (TC), orbitofrontal cortex (OFC), rostral middle frontal cortex (rMFC), fusiform cortex (FFC), insular cortex (INS), anterior cingulate cortex (ACC), posterior cingulate cortex (PCC), amygdale (AMG), hippocampus (HIP), nucleus accumbens (NAc), caudate (CAU), putamen (PUT), pallidum (PAL), thalamus (THA), brainstem (BS). Reading the mind in the eye (EYE), Movie for assessment of social cognition (MASC), Faux pas, Verbal fluency letter production (VF lp), Verbal fluency category production (VF cp), Verbal fluency semantic flexibility (VF sf), Tower test (Tower), Conner’s continuous performance test (CPT), Embedded figure test (EFT), Fragmented picture test (FPT).

**References**

1. Cselényi Z, Olsson H, Farde L, Gulyás B. Wavelet-Aided Parametric Mapping of Cerebral Dopamine D 2 Receptors Using the High Affinity PET Radioligand [ 11 C ] FLB 457. Neuroimage. 2002;60:47–60.

2. Matheson GJ, Stenkrona P, Cselényi Z, Plavén-Sigray P, Halldin C, Farde L, et al. Reliability of volumetric and surface-based normalisation and smoothing techniques for PET analysis of the cortex: A test-retest analysis using [11C]SCH-23390. Neuroimage. 2017;155:344–353.
